# Hydrogel Micro/Nanostructures for the Delivery of Oncolytic Viruses: Overcoming Limitations and Improving Efficacy

**DOI:** 10.34172/apb.025.45842

**Published:** 2025-10-11

**Authors:** Chou-Yi Hsu, Saade Abdalkareem Jasim, Jasur Alimdjanovich Rizaev, Tina Saeed Basunduwah, Vikrant Abbot, Mamata Chahar, Mohammed Asiri, Abbas Fadhel Ali, Alexey Yumashev, Ahmed Hussein Zwamel

**Affiliations:** ^1^Thunderbird School of Global Management, Arizona State University Tempe Campus, Phoenix, Arizona 85004, USA; ^2^Medical Laboratory Techniques Department, College of Health and Medical Techniques, University of Al-Maarif, Anbar, Iraq; ^3^Department of Public Health and Healthcare Management, Rector, Samarkand State Medical University, 18, Amir Temur Street, Samarkand, Uzbekistan; ^4^Department of Medical Student, University of Albatterjee College, Jeddah, Saudi Arabia; ^5^Chandigarh Pharmacy College, Chandigarh Group of Colleges, Jhanjeri, Mohali 140307, Punjab, India; ^6^Department of Chemistry, NIMS University, Jaipur, India; ^7^Department of Clinical Laboratory Sciences, College of Applied Medical Sciences, King Khalid University, Abha, Saudi Arabia; ^8^Department of Medical Laboratories Technology, AL-Nisour University College, Baghdad, Iraq; ^9^Department of Prosthetic Dentistry, First Moscow State Medical University, Russia; ^10^Medical Laboratory Technique College, the Islamic University, Najaf, Iraq; ^11^Medical Laboratory Technique College, the Islamic University of Al Diwaniyah, Al Diwaniyah, Iraq; ^12^Medical Laboratory Technique College, the Islamic University of Babylon, Babylon, Iraq

**Keywords:** Oncolytic viruses, Cancer, Immune response, Oncolytic virotherapy, Hydrogel

## Abstract

Oncolytic viruses (OVs) have attracted accumulating attention in cancer therapy owing to their ability to replicate in and kill tumor cells, resulting in the stimulation of immune responses for eradicating residual and distant malignant cells. Despite milestone achievements in the development of OVs, which led to the U.S. Food and Drug Administration (FDA) approval of talimogene laherparepvec (T-VEC) in 2015 against melanoma, there are some hurdles limiting their translation from the bench to the clinic, such as non-specific localization, host immune response against OVs and their clearance, and low efficiency as a monotherapy. Delivery of OVs with nano-biomaterials is a promising approach to address the drawback of oncolytic virotherapy. Hydrogels, with their tunable characteristics and versatile properties, offer a promising platform for the controlled release, precise delivery, and therapeutic enhancement of OVs in combination with other therapeutic agents in the treatment of cancers. This review aims to provide a deep insight into the types and development of OVs and their application in clinical trials and then will discuss the characteristics of hydrogels and how they improve the therapeutic efficacy of OVs.

## Introduction

 Conventional cancer treatment strategies, such as chemotherapy and radiotherapy, are not optimally effective due to their mechanism of action, which is based on interrupting cell division, affecting normal cells along with tumor cells.^1^ Fortunately, as our understanding of cancer has improved, including the molecular and genetic basis of cancer as well as tumor microenvironment (TME) and its cellular components and interactions, cancer treatments have far surpassed these traditional strategies and their side effects.^2^ Over the past decade, there have been several advances that have improved survival rates for some common solid tumors. One of these advances comes from developing cancer immunotherapy strategies, aiming to activate the body’s immune responses against malignant cells.^3^ As a main branch of immunotherapy, oncolytic viruses (OVs) have attracted particular attention owing to their ability to preferentially replicate within tumor cells and lyse them without affecting non-malignant ones.^4^ Currently, different viruses have been investigated as oncolytic agents for the treatment of cancers, including herpes virus, adenovirus, Newcastle disease virus (NDV), reovirus, measles virus, vaccinia virus, and poliovirus.^5^ These investigations led to the approval of four OV-based drugs for the treatment of tumors: H101, T-vec, Rigvir, and G47Δ.^6,7^ Despite these advancements and approvals, the translation of *in vitro* and pre-clinical studies to the clinic and finally to the market faces obstacles, such as immune responses against OVs, passive targeting, spread and penetration, short duration of biological activity, rapid clearance, and poor persistence in the target tissue.^8-10^ Therefore, nano-sized tools and nanomaterials have been developed to circumvent the obstacles and join OVs and nanomaterials forces against cancer.^11^

 Hydrogels are three-dimensional biomaterials with hydrophilic properties and cross-linked polymer chains, proposing them as promising structures for biomedical applications, including tissue engineering, drug screening, biosensors, bioimaging, drug delivery, and cancer immunotherapy.^12,13^ Regarding cancer immunotherapy, hydrogels not only serve as a delivery vehicle and a container of immune cells such as dendritic cells, macrophages, and chimeric antigen receptor (CAR) T-cell cells, immunomodulators, and cancer vaccines, but also provide a structure for a combination of immunotherapy and chemotherapy, called chemo-immunotherapy.^12,14^ To reach optimal therapeutic efficacy of OVs, they require escape from recognition by the host immune system as well as persistence and local delivery, for which hydrogel nano/microstructures can be considered suitable vehicles.^15^ Besides their ability to increase the concentration of the cargo at the target sites and provide controlled release, hydrogels reduce side effects and non-specific distribution of their cargo.^16^ Additionally, developing smart hydrogels that respond to external stimuli or internal ones allows them to specifically and under certain conditions release their cargo, leading to a high degree of targeting.^17^ Therefore, hydrogels with their tunable characteristics and versatile properties, offer a promising platform for the controlled release, precise delivery, and therapeutic enhancement of OVs in the treatment of cancer. Here, we will review different viruses with oncolytic activity, their mechanism of action, and their application in clinical trials. Also, we will discuss the characteristics of hydrogels as carriers of OVs and, finally, our focus will be on the state-of-the-art of hydrogel nanostructures applied in oncolytic virotherapy of cancers.

## Oncolytic virotherapy in cancer

**Figure 1:**
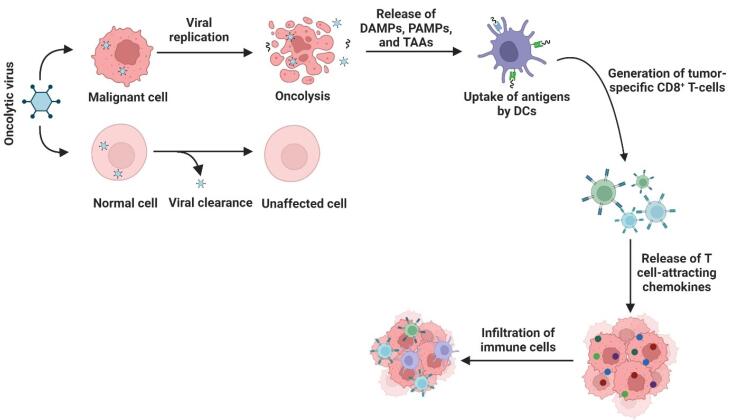
Mechanisms of action of oncolytic viruses. reated in BioRender. https://BioRender.com/ihzxzp8

 Viruses were once called “evil devils” for their role in disease, but with the rise of therapeutic viruses, especially OVs, they became “noble angels.” OVs exert their therapeutic effect through a dual mechanism (Figure 1). First, they selectively replicate in tumor cells, causing direct oncolysis. During the process of oncolysis, virus progeny will be released shortly thereafter to infect neighboring cancer cells and amplify the therapeutic response locally. Second, and importantly from an immunotherapy standpoint, destroyed tumor cells release tumor-associated antigens (TAAs), damage-associated molecular patterns (DAMPs) and pathogen-associated molecular patterns (PAMPs).^18^ This series of events initiates a strong anti-tumor immune response that can convert an overall immunologically “cold” tumors to “hot” tumors. As mentioned earlier, the immunogenicity of OVs prevents them from being neutralized, which means they can be neutralized by the host immune system before they induce a response, thereby limiting their persistence and efficacy in systemic situations. Hydrogel delivery systems are designed to circumvent this immunogenicity by protecting the virus from neutralizing antibodies and immune responses, resulting in localized delivery and prolonged release of OVs within the TME.^19^ Some OVs are naturally oncolytic in their native forms, called naturally occurring OVs (e.g., measles virus, NDV, and reovirus), whereas others require genetic modifications to exert their oncolytic functions, called genetically engineered OVs (e.g., vaccinia virus, herpesvirus, and adenovirus).^20^ Despite promising results in (pre)clinical studies, four OVs have been approved for the treatment of cancers: Rigvir (SND005) for the treatment of melanoma in Latvia (2004), Oncorine (H101) for the treatment of squamous cell cancer of the head and neck or esophagus in China (2005), Talimogene laherparepvec (T-VEC, Imlygic) for the treatment of melanoma by the U.S. FDA (2015), and Delytact (teserpaturev/G47Δ) for the treatment of malignant gliomas in Japan (2021).^21^ Table 1 summarizes some OVs in clinical trials for cancer therapy.

**Table 1:** Clinical trials of oncolytic viruses for cancer therapy.

**Virus type/name**	**Tumor type**	**Delivery route**	**Combination with**	**Phase**	**Sponsor**	**Trial No.**
HSV						
R130	Solid tumors	IT/IP	-	Early 1	Shanghai Yunying Medical Technology	NCT05886075
G207	HGG	IT	Radiotherapy	II	Pediatric Brain Tumor Consortium	NCT04482933
T3011	CRC	HAI	Toripalimab/Regorafenib	I	China Medical University, China	NCT06283303
OH2	Melanoma	IT	-	III	Binhui Biopharmaceutical Co., Ltd.	NCT05868707
MVR-C5252	Glioma	IT	-	I	Duke University	NCT06126744
RP2/​RP3	CRC	Direct or image-guided injection	Atezolizumab/ Bevacizumab	II	Replimune Inc.	NCT05733611
Adenovirus						
H101	Cervical	IT	Camrelizumab	II	Zhejiang Cancer Hospital	NCT05234905
TILT-123	Ovarian	IT/IP	Pembrolizumab	I	TILT Biotherapeutics Ltd.	NCT05271318
H101	ICC	IT	HAIC	IV	Beijing Tsinghua Chang Gung Hospital	NCT05124002
CG0070	IR-NMIBC	N/A	TURBT	III	CG Oncology, Inc.	NCT06111235
KD01	Cervical	IT	-	I	Tongji Hospital	NCT06552598
AdAPT-001	Sarcoma/ Solid tumors	IT	-	II	EpicentRx, Inc.	NCT04673942
VACV						
OVV-01	Solid tumors	IT	Pembrolizumab/ Atezolizumab	I	North China Petroleum Bureau General Hospital	NCT04787003
TBio-6517	Solid tumors	IT/IV	Pembrolizumab	I/II	Turnstone Biologics, Corp.	NCT04301011
hV01	Pancreatic	IT	-	II	Hangzhou Converd Co., Ltd.	NCT07006077
Olvi-Vec	Ovarian	IP	Platinum-doublet/ Bevacizumab	III	Genelux Corporation	NCT05281471
IDOV-Immune	Solid tumors	IV	-	I	ViroMissile, Inc	NCT06910657
RGV004	BCL	IT	-	I	Second Affiliated Hospital, School of Medicine, Zhejiang University	NCT04887025
MV						
MV-s-NAP	Breast	IT	-	I	Mayo Clinic	NCT04521764
MV-NIS	Myeloma	IV	Cyclophosphamide	II	University of Arkansas	NCT02192775
Reovirus						
Pelareorep	TNBC	IV	INCMGA00012	II	Mridula George, MD	NCT04445844
Pelareorep	MM	IV	Bortezomib/ Pembrolizumab	I/II	University of Southern California	NCT05514990

HSV, herpes simplex virus; IT, intratumoral, IP, intraperitoneal; HGG, high grade glioma; CRC, colorectal cancer; HAI, hepatic artery infusion; ICC, intrahepatic cholangiocarcinoma; HAIC, hepatic arterial infusion chemotherapy; IR-NMIBC, intermediate risk non-muscle invasive bladder cancer; TURBT, transurethral resection of bladder tumor; N/A, not available; VACV, vaccinia virus; IV, intravenous; BCL, B-cell lymphoma; MV, measles virus; TNBC, triple-negative breast cancer; MM, multiple myeloma.

###  Herpes simplex virus

 The only FDA-approved OV in cancer therapy is a herpes simplex virus (HSV-1)-derived agent, called T-VEC, in which viral infected cell protein (ICP) 34.5 and ICP47 genes were deleted and the human granulocyte-macrophage colony-stimulating factor (GM-CSF) gene was inserted into the genome of the virus. ICP34.5 deletion attenuates the neurovirulence capacity and improves tumor-selective replication of the virus, whereas ICP47 deletion declines virally mediated inhibition of tumor-associated peptides and improves antigen presentation. Moreover, the unique short 11 gene (US11) translocation enhances the tumor-lytic activity of the virus and GM-CSF insertion increases APC recruitment.^22^ In addition to T-VEC, other HSV-based OVs have been developed and translated into clinical trials, such as G207, HSV-1716, rQNestin34.5, G47Δ, OH2, ONCR-177, and RP1.^23,24^

###  Adenovirus

 Two main approaches have been used to develop oncolytic adenoviruses (Ads): deletions in the *E1A* and *E1B* gene regions and replacement of the native E1 promoter with tumor-specific promoters.^25^ Oncorine, an Ad5-based virus with deletions in the *E1B* (55 kDa) and *E3* gene fragment (78.3-85.8 mu), is the first approved OV for clinical use. Deletion of E1B-55KD leads to selective replication of the virus within tumor cells with dysfunction of the P53 pathway, whereas deletion of the *E3* gene fragment enhances the safety of the virus.^26^ The promising results of Oncorine inspired the entry of other oncolytic Ads into clinical trials, such as LOAd703, CG0070, OBP-301 (Telomelysin), DNX-2401, TILT-123, VCN-01, *etc*.^27^

 While OAds show great potential, their delivery is still a critical hurdle to overcome. When administered systemically, they are commonly sequestered rapidly by the liver and spleen and neutralized by pre-existing anti-Ad antibodies, even at high viral doses.^28^ To address this, simple non-viral delivery vectors have been investigated, including lipid nanoparticles (LNPs) and polymeric nanoparticles, which can protect OAds from neutralizing antibodies and can easily be surface functionalized for active targeting.^29^ However, nanoparticles tend to have a small loading capacity, are associated with the potential for complement activation and rapid clearance, and are unable to be localized. Instead, in our hands, hydrogels appear to be a much better platform for local or regional delivery. When therapeutic agents are encapsulated in a hydrogel, this results in the formation of a localized depot that allows for sustained release of the cargo over days or weeks, while keeping its concentration high directly within the TME.^10,30,31^ This localized delivery really helps to circumvent systemic toxicity and is most readily applied to surgically accessible tumors or in regard to adjuvant therapy/biologic after tumor resection.^32,33^

###  Vaccinia virus

 Several advantages make vaccinia virus (VACV) a favorable agent for oncolytic virotherapy: replication within the cytoplasm that reduces its mutagenesis possibility, an extensive and long history in humans that guarantees its safety, such as the smallpox vaccine, the capacity to carry long segments of foreign DNA, the ability to infect a wide range of cells, a natural tropism toward cancer cells, and a rapid replication and lytic rate to induce inflammation and immune responses.^34,35^ Although unmodified VACVs could lyse cancer cells, their application in the clinic is limited because of reported side effects, including fatal neuroencephalitis and hepatitis; thus, genomic modifications have been made to improve clinical efficiency by reducing immunogenicity and toxicity as well as enhancing tumor selectivity and therapeutic outcomes.^36^ For example, the *J2R* gene deletion, which encodes thymidine kinase (TK), is the most common modification during the development of oncolytic VACVs, leading to tumor-selective replication of the virus and reduced pathogenicity.^37^ JX-594 (Pexa-Vec®) is an oncolytic VACV in which the *TK* gene was inactivated, whereas GM-CSF and β-galactosidase were inserted.^38^ In addition to JX-594, other developed oncolytic VACVs also were tested in clinical trials, such as GL-ONC1 (GLV-1h68), TroVax, TG4010, PROSTVAC, PANVAC, rV-B7.1, MVA-5T4, and IN rVV.^36^

###  Measles virus

 Hemagglutinin (H) and fusion (F) proteins of the virus envelope are involved in its attachment to the target cell and fusion, while CD150/SLAM, CD46, and nectin-4 are considered the main receptors of measles virus (MV). Due to the upregulation of CD46 on the malignant cells, MV exhibits oncotropism and induces cell death by inducing fusion of cells and the formation of syncytia.^39^ Engineering MVs by mutating the receptor binding sites targeted them toward EGFRvIII-expressing glioblastoma,^40^ folate receptor (FR)-α-expressing ovarian cancer,^39^ and CD20-expressing B-cell malignancies,^41^ whereas fusing targeting moieties such as antibody single-chain variable fragments (scFv),^42^ cystine knot proteins,^43^ and integrin-binding peptides^44^ has also been developed to direct oncolytic MVs toward tumor cells. To monitor oncolytic MV *in vivo*, the virus is engineered to express sodium iodide symporter (NIS), allowing their tracking using radiotherapy with iodine-131 (^131^I), 99m-technetium uptake, and γ-camera imaging of ^123^I.^45^ Various types of oncolytic MVs have entered clinical trials owing to their promising results in *in vitro* and *in vivo* studies, such as MV-s-NAP for breast cancer, MV-NIS for malignant pleural mesothelioma and multiple myeloma, MV-CEA and MV-NIS for ovarian epithelial cancer or primary peritoneal cancer, and TMV-018 for tumors of the gastrointestinal tract. For instance, a phase I clinical trial using MV-CEA in 22 glioblastoma patients revealed that MV-CEA improves overall survival and has no dose-limiting toxicity. Intratumoral administration of MV-CEA was also associated with pro-inflammatory remodeling of the tumor milieu and increased infiltration of CD8^+^T-cells within the TME.^46^ A vaccine, named MV-s-NAP, against metastatic breast cancer is under investigation (NCT04521764), in which MV encodes *Helicobacter pylori* neutrophil activating protein (NAP) to increase innate immune recruitment into the TME.

###  Other OVs

 Besides the mentioned viruses, other ones have also been used as OVs, including reoviruses, protoparvoviruses, polioviruses, and NDV. Reolysin (Pelareorep) is an unmodified reovirus that was investigated in clinical trials either as monotherapy or in combination with other therapeutic agents, such as chemotherapy and immune checkpoint inhibitors. For instance, Noonan et al studied the combination of reolysin with carboplatin and paclitaxel for the treatment of metastatic pancreatic adenocarcinoma in a randomized phase II trial. Although reolysin exhibited a safe profile, it did not improve the progression-free survival (PFS) compared with monotherapy with chemotherapeutic agents. They also found that a chemotherapy combination is critical to improve immunological outcomes of reolysin.^47^ Furthermore, reolysin could suppress both CTL and NK cell responses owing to the upregulation of immune checkpoint molecules.^47,48^ The smallest OV under clinical investigation is H-1PV (ParvOryx), belonging to the *protoparvovirus* (PV) genus, with a 25 nm diameter.^49^ Due to its small size, H-1PV could cross the blood-brain/tumor barrier in humans and concentrate in the brain/tumor. A phase I/IIa clinical trial using H-1PV revealed that the virus is safe and could trigger an immunogenic stimulus in glioblastoma patients, including CTL infiltrations.^50^ Intravenous administration of H-1PV to seven patients with pancreatic ductal adenocarcinoma and at least one liver metastasis also confirmed its safety. H-1PV was not only present in the tumor samples, but also enhanced the levels of pro-inflammatory interleukins (such as IFN-γ, IL-8, IL-9, and IL-12), pro-migratory cytokines (such as CXCL9), and T-cells.^51^ To date, various types of oncolytic polioviruses have been developed against cancers, such as PVSRIPO and PV-1. More than ten clinical trials tried to investigate the safety and efficacy of PVSRIPO in the treatment of glioblastoma, melanoma, and breast cancer either as monotherapy or in combination with other agents (NCT04479241, NCT04577807, and NCT03564782). A phase 1 trial with PVSRIPO, a recombinant type I poliovirus (Sabin) vaccine carrying an internal ribosomal entry site (IRES) of human rhinovirus type 2, which uses CD155 to target malignant cells, in melanoma patients showed no serious adverse events with encouraging antitumor responses.^52^ In addition to the higher expression in solid cancers, CD155 upregulation on APCs activates tumor-associated APCs in the TME and augments type I/III IFN production, leading to tumor growth suppression.^53,54^ A phase II study of PVSRIPO plus pembrolizumab for recurrent glioblastoma (NCT04479241) showed a median overall survival of 12.5 months, which is favorable when compared to historical controls and suggests synergistic effects with immune checkpoint blockade.^55^ Likewise, pelareorep (reovirus) has shown promise in combination therapies. According to a phase I/II study, patients with metastatic pancreatic adenocarcinoma received pelareorep plus atezolizumab plus chemotherapy and showed favorable modulation of immune response in the TME, as well as an encouraging objective response rate.^56^ Taken together, unmodified and genetically engineered viruses could act as oncolytic agents to selectively target tumor cells.

## Hydrogels: structures and characteristics

**Figure 2:**
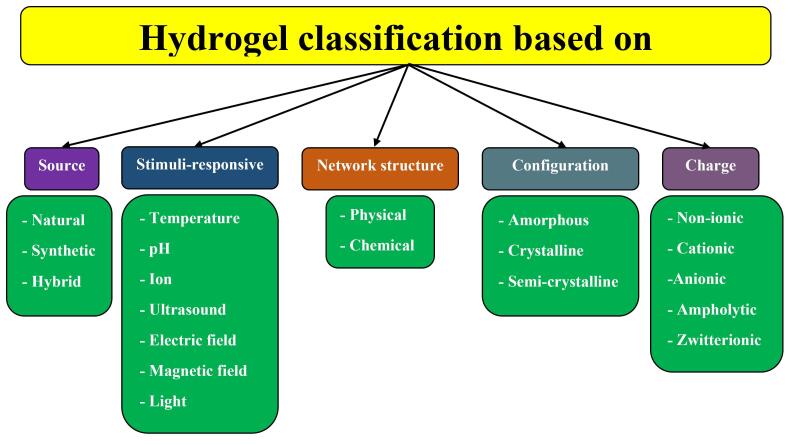
Classification of hydrogels according to various characteristics

 According to the descriptions, hydrogels contain various properties, including hydrophilicity, polymeric networks, three-dimensional, water-swollen, and cross-linked structures.^57^ Therefore, hydrogels can be classified into varied groups based on their diverse characteristics (Figure 2): 1) *Source:* The source of hydrogels can be natural, synthetic, or hybrid (combining natural and synthetic polymers). Natural sources of hydrogels come from collagen, gelatin, dextran, alginate, lignin, cellulose, agar-agar, and chitosan, whereas synthetic hydrogels are made from polymers such as N-vinyl 2-pyrrolidone (NVP), ethylene glycol (EG), vinyl acetate (VAc), methacrylic acid, hydroxy methyl methacrylate (HEMA), and ethylene glycol dimethylacrylate (EGDMA).^58^ Although natural-based hydrogels are bioactive, biodegradable, and biocompatible, their allergenicity, immunological risks, and weak mechanical strength and stability are considered their limitations.^59^ On the other hand, hybrid hydrogels take more attention in biomedicine due to their well-defined structures and higher capacity of water absorption, strength, and durability.^60^ 2) *Stimuli-responsive:* Hydrogels, according to various applications, can respond to different stimuli, including temperature, pH, and ionic strength. These hydrogels are also called smart hydrogels. For instance, thermoresponsive hydrogels undergo a phase transition from a sol phase to a gel phase due to temperature increase and return back to the liquid phase by decreasing the temperature to a certain range.^61^ 3) *Network structure:* Another criterion for hydrogel classification is based on cross-linking networks: physically cross-linked hydrogels (reversible gels) and chemically cross-linked hydrogels (permanent gels). Due to not using cross-linking agents and ease of production, physically cross-linked hydrogels have gained significant attention.^57^ 4) *Configuration:* Hydrogels also can be classified into three groups according to their configuration or structure: amorphous, crystalline, and semi-crystalline. Crystalline hydrogels contain a strongly packed polymer network structure with the order of crystallization, whereas amorphous ones consist of a random network at the molecular level. Additionally, semi-crystalline hydrogels are a mixture of amorphous and crystalline structures.^59^ 5) *Charge:* Based on the electrical charge on the cross-linked network, hydrogels are categorized into four classes: nonionic (neutral), ionic (cationic and anionic), zwitterionic (polybetaines), and amphoteric electrolyte (ampholytic). Nonionic hydrogels contain no charge on the polymer backbone or side groups, whereas cationic hydrogels carry positively-charged functional groups, such as thiol and amines, and anionic hydrogels consist of negatively-charged functional groups in their structures, such as carboxyl and sulfonyl.^62^ Moreover, zwitterionic hydrogels contain both cationic and anionic groups, while amphoteric ones carry both acidic and basic groups.^60^

## Hydrogel for delivery of oncolytic viruses

###  Advanced hydrogel platforms for delivery

 Progressions in nanotechnology and polymer sciences led to the development of “smart” hydrogels for the delivery of therapeutic agents, in which hydrogels release their cargo in response to external stimuli, instead of conventional and “dumb” hydrogels. Traditional “dumb” gels can expand or contract due to osmotic pressure; however, their responsiveness tends to be inefficient, resulting in inaccurate drug release and restricted control over dosage timing, whereas “smart” gels, known as stimuli-responsive hydrogels, are designed to react to different stimuli including magnetic fields, electromagnetic radiation, pH levels, temperature, or the existence of particular biological elements.^63^ For example, the mechanism of action of thermosensitive hydrogels is based on phase transition (from sol to gel) at the critical solution temperature in response to temperature changes. The lower critical solution temperature (LCST) and upper critical solution temperature (UCST) are major properties of thermosensitive hydrogels as they describe the phase transition of these materials on temperature change. LCST is defined as the temperature below which a thermosensitive hydrogel is soluble (in a sol state), and above the temperature were hydrophobic interactions or the collapse of the polymer chains cause the formation of a gel (gelling state). The transition from so hydrated state to a gel state is a result of the balance between the properties of hydrophilic and hydrophobic segments chemically or physically incorporated in the polymer. UCST is above the temperature where a thermosensitive hydrogel is soluble (sol state), and below which a hydrogel forms a gel due to intermolecular interactions, such as hydrogen bonding or ionic interactions.^64,65^ LCST-based systems have become popular designs in the biomedical field because their transition temperatures can be designed to match ~32-37 °C (physiological temperature).^66^ Considering the source of their composition, thermosensitive hydrogels are divides as natural biodegradable polymers, including gelatin, cellulose, and chitosan, and synthetic polymers, such as polyethylene glycol (PEG) and poly(N-isopropyl acrylamides) (PNIPAM).^65^ Owing to its LCST ~32 °C, PNIPAM remains a suitable polymer for biomedical applications.^67^ Other stimuli-responsive hydrogels are enzyme-responsive hydrogels (ERHs) that change structure in response to enzyme activities, allowing existing properties of the materials to be altered based on specific biomolecular signals.^68^ For example, if an ERH were responsive specifically to matrix metalloproteinases (MMPs), which are often overexpressed in cancer, then by using the ERH, the release of the therapeutic agent and degradation of the biomaterial could be controlled where they are both released locally because of the local overexpression of MMPs, thus enhancing efficacy of the therapeutic agent and reduced off-target effects.^69^ Enzyme sensitive hydrogels proceed a bit differently, but the primary functionality will be through site-specific degradation or controlled release of different therapeutics by using specific linkers to therapeutically active and elastomeric materials. There are primary three different modes of enzyme mediated degradation used in ERHs: (1) Matrix degradation, in which enzymes begin degrading crosslinkers causing the hydrogel to disintegrate and release the encapsulated therapeutics; (2) Cleavage of covalently attached therapeutics, either tethered by enzyme cleavage linker or chemically; where therapeutic remains attached to the hydrogel until the enzymes activate the linkers, controlled release can then occur; (3) Conformational change by altering the “swelling” or “hydrophilicity” of the hydrogel and the rates of diffusions of the drug can be imparted by the enzymatic activity.^70,71^ Stimuli-responsive hydrogels can also be classified into two groups based on response to stimuli: single-stimulus responsive hydrogels and multi-stimuli responsive hydrogels. Compared with single-stimulus responsive hydrogels, multi-stimuli responsive hydrogels have some advantages because of their capability to respond to various external stimuli at the same time or in sequence, including improved reliability owing to their higher specificity, enhanced versatility, greater sensitivity, more stable, and synergistic effects.^72^ Design strategies focus on functionalizing stimuli-sensitive moieties into polymer networks for desirable properties. Stimuli-responsive polymer networks can be generated by covalent crosslinking, using supramolecular interactions (e.g., host-guest chemistry) or creating interpenetrating polymer networks (IPNs).^73^ This results in materials with tunable response characteristics. Biocompatible responses can also be enhanced by ‘borrowing’ functions from synthetic polymers built from stimuli-responsive monomers. Typically, a polymer would have a synthetic polymer, such as PNIPAm, which serves as a thermoresponsive component, along with groups that support responses at the pH level (for example, acrylic acid) or that respond to redox (for example, disulfide bonds).^74^ Natural polymers that also serve as conductive elements, such as chitosan or hyaluronic acid, are used as the basis of a hybrid construct with multi-stimuli capabilities (e.g., fibre aniline oligomers or nanoparticles, including magnetic Fe3O4).^74,75^ More advanced constructs can be developed that use complex supramolecular assemblies built from β-cyclodextrin (β-CD) containing azobenzene or ferrocene as guests, giving rise to materials with photo- and redox-responsiveness, allowing potentially reversible sol-gel behavior.^76^ Other advanced techniques, such as layer-by-layer (LbL) assembly, enable the production of multilayer films with the potential to control pH and temperature response mechanisms.^77^

 Another promising alternative to conventional hydrogels is self-healing ones. In contrast to conventional hydrogels that have restricted mechanical stability and deforming characteristics due to mechanical forces, self-healing platforms have long-term stability and good reliability because they can restore their original function, structure, and shape.^78,79^ This self-repairing feature usually depends on restoring molecular interactions in a moisture-rich microenvironment once the hydrogel has encountered external forces or harm.^78^ These hydrogels can be linked through dynamic covalent bonds or noncovalent interactions, allowing them to heal and reform via the dynamic balance between the separation and reassociation of these interactions. Upon implantation or injection, self-healing hydrogels can endure *in vivo* structural changes without suffering damage. Self-healing hydrogels are usually water-dense polymers characterized by a porous framework. As a result, water-soluble bioactive agents dissolve effectively in hydrogels and adhere within porous structures and release in the target tissue.^78,80^ Recent developments have carefully explored new compositions to expand functions, including functionalized chitosan-based self-healing hydrogels, which take advantage of the natural polymer’s biocompatibility and contain dynamic imine linkages for rapid healing in wound dressings.^81^ Other innovative designs include photo-regulated self-healing hydrogels with hollow nanoparticles, which can increase the modulus of the material with light so that Young’s modulus can be up to synchronous growth in tissue repair applications.^82^ Recently cellulose based hydrogels have emerged as a sustainable and tunable swelling dynamics with molecular network architectures that respond to physicochemical stimuli with numerous potential uses in delivery applications and in regenerative medicine.^83^ Developments in additional directions should address challenges in mechanical durability and specificity and position self-healing hydrogels as versatile platforms for different biomedical applications for which they could be used for targeted therapy.

 Nanogels, nanoparticle-based hydrogels, are another innovative and promising advanced platform. They consist of interconnected hydrophilic polymers and water, with an average diameter of approximately 100 nm. They possess a significant water content, an extensive specific surface area, and robust stability. Nanogel-centered systems are engineered to ensure the cargo maintains an extended circulation half-life within the body and can effectively deliver it to intended sites in biomedical applications. Additionally, nanogels can be designed to respond to environmental factors; these are referred to as stimulus-responsive or environmentally responsive nanogels.^84,85^ The characteristics of the nanomaterials within the hydrogel influence the type and functionality of stimuli-responsive hydrogels. Moreover, the incorporation of nanomaterials into hydrogels enhances their injectability and shear-thinning characteristics. The porous microstructure of hydrogels and the diverse interactions between nanomaterials and hydrophobic polymer chains allow nanomaterials to influence the rheological behavior of hydrogels through distinct interactions, leading to enhanced viscoelastic properties. A proper distribution of nanomaterials within hydrogels leads to enhanced biocompatibility and improved functionality of nano-crosslinked dynamic hydrogels.^86^ Molecular architectures of nanogels can utilize dynamic crosslinking approaches to achieve tunable properties, chemical crosslinking via click chemistries (thiol-maleimide Michael additions or strain-promoted azide-alkyne cycloadditions) to form redox-degradable networks, or physical self-assembly with electrostatic interactions and hydrogen bonding via reversible structures.^87^ Many fabrications of stimuli-responsive designs have also progressed, including pH-sensitive nanogels, which use ionizable groups (carboxylic acids or amines) to induce swelling and drug release within acidic TMEs, redox-responsive nanogels utilizing disulfide bonds, which are destructed intracellularly in glutathione-rich environments, and thermo-responsive polymers, with examples using PNIPAM, which transition around body temperature to allow controlled release.^88^ As shown in the multi-stimuli designs, there are also byproducts that offer combinations of mechanisms such as pH/redox dual-responsive nanogels from hyaluronic acid crosslinked by cystamine, which execute targeted delivery of cargo while promoting on-target delivery and reducing off-target effects.^89^ New compositional innovations expand the versatility of nanogels, including natural polymers, such as hyaluronic acid, alginate, and chitosan with the intent of allowing for some biocompatibility, synthetic polymers, such as PNIPAM and PEG leading to tunable hydrophilicity, and hybrid nanogels utilizing nanomaterials, such as gold-silver-cuprous oxide nanoparticles (Au-Ag-Cu2O NPs) with potential photothermal effects.^87^ For example, nucleic acid-based nanogels, made by DNA hybridization or creating DNA origami structures, are extremely versatile, are very good at loading drugs such as siRNA and mRNA, have potential for a high cargo and their programmed structures protect against nucleases, static charge, and enable size-targeted nanoparticle methods of gene therapy.^89^ It is worth noting that using microfluidics in nanogel synthesis is one of the recent advancements in this area. Microfluidics provides a precise and powerful tool for real-time monitoring and efficient polymerization that circumvents scalability challenges and guarantees uniform particle production.^90^

###  Hydrogels deliver oncolytic viruses to the tumor microenvironment

**Figure 3:**
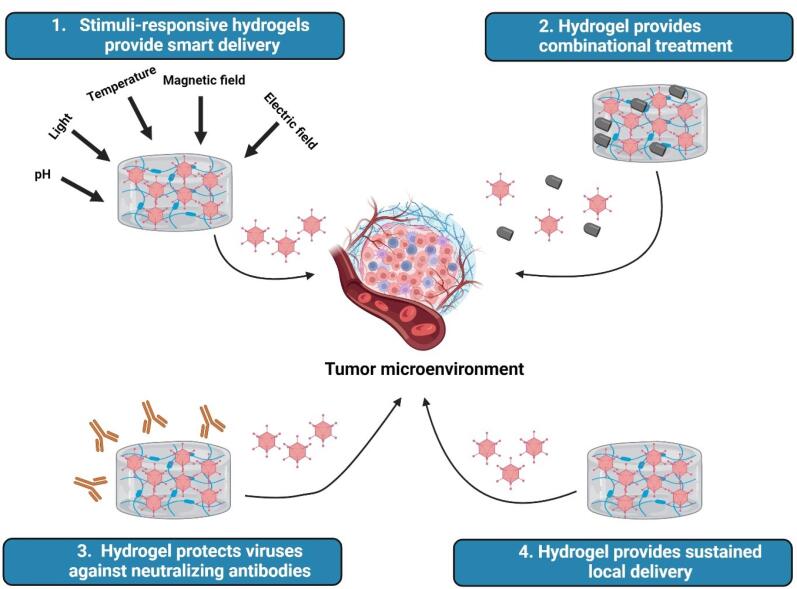
Hydrogel and oncolytic virotherapy. Hydrogel improves the therapeutic effects of oncolytic viruses by providing smart delivery, providing a scaffold for combinational therapy, protecting the virus against neutralizing antibodies, and providing sustained local delivery. Created in BioRender. https://BioRender.com/t41rl18

 Despite great attention to OVs, they need to circumvent some limitations to revolutionize the cancer treatment market. For example, OAds require high doses and repeated administration to show promising anti-cancer effects due to poor persistence of the virus in a target tissue, short duration of gene expression with therapeutic goals, and virus-specific immune responses within the host.^91,92^ As shown in Figure 3, hydrogel-based micro/nanomaterials can overcome these challenges because they protect their cargo from the host’s harsh environments and retain their cargo over an extended time in the target tissue with high concentration.^93,94^ Moreover, hydrogels provide sustained local delivery of OVs into the TME to improve therapeutic outcomes.^10,95^ Owing to its responsiveness to the TME, including matrix metalloproteinase-2 (MMP-2), MMP-9, and collagenase, Jung et al designed a gelatin-based hydrogel for sustained delivery of tumor necrosis factor-related apoptosis-inducing ligand (TRAIL)-expressing OAd (OAd-TRAIL) into the TME. They found that the stiffness of hydrogel affects the kinetics of the release of OAds, proposing that 3380-Pa gel provides a more tightly regulated (enzyme-dependent) release profile compared with 50-Pa gel. Surprisingly and in contrast to *in vitro* experiments, they reported that the 50-Pa gel-mediated delivery system leads to more effective release of OAd and exhibits anti-tumor activities in Syrian hamsters compared with the 3380-Pa gel. Encapsulation of OAd-TRAIL into the hydrogel system also prolongs virus persistence and enhances viral localization and accumulation into the TME due to the time-dependent degradation of the hydrogel. Moreover, the OAd-TRAIL/gel system displayed lower liver accumulation (15.4-fold), indicating lower hepatotoxicity, and reduced innate immune responses (IL-6 and TNF-α) as well as adaptive immune responses (neutralizing antibodies) against OAd.^19^ The potential reason for the inconsistency between the *in vitro* and *in vivo* results could be explained as follows: 1) Due to better accessibility of enzymes to cleavage sites and looser polymer network, MMPs may degrade softer hydrogels faster than stiff ones *in vivo,* which leads to burst release of OVs and potent anti-tumor immune responses in softer gels may stem from better infiltration of immune cells into the TME.^96,97^ In addition to collagenase, which was used in the *in vitro* conditions of this study, the TME has a complex environment with esterases, glycosidases, and proteases activities,^98^ all of which can affect the kinetics of cargo release and hydrogel degradation. This difference in heterogeneity of enzymatic activity between the TME and *in vitro* conditions can lead to different results. Furthermore, infiltration of immune cells occurs better in softer hydrogels,^99,100^ which degrade faster, subsequently, resulting in release of antigens, PAMPS, and DAMPs from lysed malignant cells, priming the “cold” TME to “hot” one. This rapid activation of immune responses might lead to potent anti-tumor activities *in vivo*. In another study, Le et al developed a dual temperature- and pH-responsive physically cross-linked hydrogel-based structure for long-term and efficient delivery of OAd. This system rapidly formed a gel at the TME or body condition, whereas it was in sol form at room temperature and pH = 9. The sol/gel transition of the polyurethanes (PUSMA)-based hydrogel can allow it to be injectable for delivery purposes. The physically cross-linked hydrogel protected OAds from the environment and retained their anti-tumor therapeutic effects for a long time compared to naked OAds, leading to robust cytotoxicity against malignant cells even for 11 days.^101^ In addition to the temperature- and pH-responsive hydrogels, redox-responsive hydrogels have also been designed for OV delivery in which crosslinking based on disulfide bonds could be cleaved by high concentrations of glutathione (GSH), a reduction agent, at the intracellular or tumor region. In this regard, Deng et aldesigned a nano-dimensional redox-responsive hydrogel, 300-400 nm in diameter and -13 mV zeta potential, for the encapsulation and delivery of two model OVs, echovirus Rigvir® ECHO-7 (RNA virus) and Ad [I/PPT-E1A] (DNA virus), and to protect them against circulatory antibodies. The redox-responsive OVs/nanohydrogel systems not only maintained stability in normal physiologic conditions, but also provided the controlled release of OVs and induced cell lysis of malignant cells.^102^

 Hydrogel micro/nanostructures also facilitate combinational therapy using OVs and other therapeutic agents. During the treatment profile, high-dose repeated administration of both OVs and therapeutic agents is applied to compensate for their inactivation, proposing the development of co-delivery systems, such as hydrogel, to conquer these limitations. To prolong and enhance the therapeutic ability of OAd armed with IL-12 and IL1-5 (CRAd-IL12-IL15) and cytokine-induced killer (CIK) cells in a single local injection, Du et al encapsulated both therapeutic agents into a gelatin-based hydrogel. The CRAd-IL12-IL15 + CIKs/gelatin structure not only reduced the spreading of high-dose OAds and CIK cells from the vaccination site to the non-target tissues, such as the liver, but also attenuated immune responses against Ad and maintained sustained release of both therapeutic agents.^103^ Another study combined IL-12 and GM-CSF-expressing OAd with DCs using a biodegradable hydrogel to ameliorate immunosuppressive TME and impede their rapid inactivation and dissemination. Compared with OAd + DCs or a single treatment, the OAd + DC/gel system (1 × 10^10^ VP and 1 × 10^6^ DC cells) activated both exogenous and endogenous DCs and enhanced their infiltration into draining lymph nodes. Additionally, the OAd + DC/gel system increased the number of tumor-specific IFN-γ-secreting immune cells and presented considerably greater tumor growth inhibition than the OAd + DC-vaccinated group. These beneficial results of the OAd + DC/gel system returns to higher population of OAds and DCs within the TME owing to release kinetics which prolonged intratumoral retention of both therapeutic agents. Retention of OAds and DCs by the hydrogel system in the tumor tissue augments the level of cytokines and finally increases infiltration of immune cells and augments DCs maturation.^104^ In hydrogel-based delivery platforms, especially in co-delivery systems, determining a release profile for achieving an ideal therapeutic advantage is a major challenge. The development of mathematical models, such as ordinary differential equations (ODEs), could be helpful in predicting and optimizing treatment protocols as well as determining optimal doses and schedules.^105-107^ ODE models simulate the multifaceted interactions of therapeutic agents with tissues and cells to determine the effects of treatment on cells, tumor growth, personalized medicine, immune cell responses, immune evasion, resistance management, and treatment schedule.^108,109^ Indeed, ODE models use temporal dynamics of cell population and response to treatment for representing the rate of change over time.^110^ In this regard, Jenner et al used a mathematical model (ODEs) to optimize the release profile in combinational therapy (OAd and immature DCs) loaded onto a hydrogel system in interaction with immune cells and tumor cells. Their modeling revealed that (1) different immune and virus characteristics are tumor cell-specific, (2) the division of resources between anti-viral and anti-tumor immune responses potentiates fighting against tumors in combinational treatments, (3) the significantly reduced tumor volume stems from an initial burst release of OAd from the hydrogel delivery system, which infects and stimulates an immune response, followed by a sustained constant release of DCs, and (4) additional complexity in the hydrogel-release profile is not vital for any improvement in the treatment outcome. In this model, they included the release of both OVs and immature DCs as well as the roles of uninfected malignant cells in stimulating immune cells. The final ODE system is as follows:

 1) 𝑑𝑈/𝑑𝑡 = 𝑟 log (𝐿/𝑈)𝑈 – 𝛽𝑈𝑉/𝑇−𝜅𝐾𝑈/𝑇,

 2) 𝑑𝐼/𝑑𝑡 = 𝛽𝑈𝑉/𝑇 − 𝑑_𝐼_𝐼 – 𝜅𝐾𝐼/𝑇,

 3) 𝑑𝑉/𝑑𝑡 = 𝑢_𝑉_(𝑡) − 𝑑_𝑉_𝑉 + 𝛼𝑑_𝐼_𝐼,

 4) 𝑑𝐷_𝑆_/𝑑𝑡 = (1−𝑓)𝑢_𝐷𝐶_ (𝑡) – 𝑠_𝐴𝑈_𝐷_𝑆_𝑈 − 𝑠_𝐴𝐼_𝐷_𝑆_𝐼 − 𝑑_𝑆_𝐷_𝑆_,

 5) 𝑑𝐷_𝐿_/𝑑𝑡 = 𝑓𝑢_𝐷𝐶_ (𝑡) −𝑠_𝐴𝑈_𝐷_𝐿_𝑈 − 𝑠_𝐴𝐼_𝐷_𝐿_𝐼 − 𝑑_𝐿_𝐷_𝐿_,

 6) 𝑑𝐴_𝐼_/𝑑𝑡 = 𝑟_𝐴𝐼_𝐼 − 𝑠_𝐴𝑈_𝐴_𝐼_𝑈 − 𝑠_𝐴𝐼_𝐴_𝐼_𝐼 – 𝑑_𝐴𝐼_𝐴_𝐼_,

 7) 𝑑𝐴_𝑀_𝑑𝑡 = 𝑠_𝐴𝑈_𝑈 (𝐴_𝐼_ + 𝐷_𝐿_ + 𝐷_𝑆_) + 𝑠_𝐴𝐼_𝐼 (𝐴_𝐼_ + 𝐷_𝐿_ + 𝐷_𝑆_) − 𝑑_𝐴_𝐴_𝑀_,

 8) 𝑑𝐻/𝑑𝑡 = 𝑠_𝐻_𝐴_𝑀_ − 𝑑_𝐻_𝐻,

 9) 𝑑𝐾/𝑑𝑡 = 𝑠_𝐾𝐻_𝐻 + 𝑠_𝐾𝐴_𝐴_𝑀_ − 𝑑_𝐾_𝐾.

 where *t* is time (days); *U* and *I* are the uninfected and infected tumor cells, respectively; *V* is free virus particles; *D*_S_ and *D*_L_ are short-lived and long-lived immature DCs, respectively; *A*_I_ and *A*_M_ are immature and mature APCs, respectively; *K* is killer T-cell, and *H* is helper T-cell. Furthermore, they presented the 𝑇 = 𝑈 + 𝐼 + 𝐷_𝐿_ + 𝐷_𝑆_ + 𝐴_𝐼_ + 𝐴_𝑀_ + 𝐻 + 𝐾 equation for the total cell [population within the TME.^111^

 To overcome immunosuppressive TME, Zhuang et al co-loaded an oncolytic HSV-1 (1 × 10^7^ pfu) and Navoximod (120 μg), an indoleamine 2, 3-dioxygenase 1 (IDO1) inhibitor, into 2% silk-hydrogels (V-Navo@gel). In the V-Navo@gel system, the intratumoral injection of hydrogel reduces the toxicity of the oncolytic HSV-1 by restricting the virus dissemination. Furthermore, the upregulation of IDO1 is inhibited by the release of Navoximod from the V-Navo@gel, leading to the reshaping and reversal of immunosuppressive TME and inhibition of tumor growth.^112^ While the clinical translation of such a system holds enormous promise, its challenges include the scale-up production of the GMP-grade silk hydrogels and the variability of the TME composition and expression of IDO1 from one patient to another. Future research could enhance specificity by functionalizing the silk fibroin polymer with targeting ligands such as RGD (arginine-glycine-aspartic acid) peptides. In addition to providing scaffolds for OVs and cancer immunotherapy approaches, hydrogels facilitate the co-encapsulation and delivery of OVs with other tumoricidal agents. For instance, Qiao et al assessed the anti-tumor effects of injectable pH-degradable polyvinyl alcohol (PVA) microgels co-encapsulated with OAd and bromodomain and extra-terminal (BET), JQ1, using microfluidics. The cargos of the PVA microgel were released due to cleavage of vinyl ether acrylate (VEA)-functionalized PVA in a mildly acidic environment and the co-loaded microgel exhibited significant cytotoxicity against A549 lung cancer cells. Furthermore, the addition of JQ1 treatment to OAd in microgels enhanced the Ad replication *in vivo* and reversed the immune suppression by inhibiting PD-L1 expression, leading to tumor inhibition.^15^ It is worth noting that OV-loading hydrogel beads and its combination with transcatheter arterial embolization (TAE) is a sufficient approach for targeting malignant cells. In this combinational approach, OV-loading hydrogel beads act as an effective embolic system for transcatheter arterial viroembolization (TAVE) in the treatment of hepatocellular carcinoma. Also, it provides sustained release of OVs for an extended period and prevents virus accumulation within normal tissues.^113^

## Conclusion

 OVs, as preferentially lysing tumor cell agents, have gained accumulating attention due to promising therapeutic potential in (pre)clinical studies. Nevertheless, their anti-tumor activity is hindered by various factors, including non-specific targeting in systematic delivery, neutralization by pre-existing antibodies, immunosuppressive TME, and physical barriers. To address these limitations, different delivery systems have been developed. Among them, hydrogels have unique advantages that make them more appealing, such as biodegradability, biocompatibility, the ability to respond to multiple stimuli, high loading capacities, and low toxicity. Compared with other delivery platforms, such as nanoparticles and liposomes, OV delivery using hydrogels presents several advantages. In contrast to nanoparticles and liposomes that are suitable for systemic delivery, hydrogels represent another class of effective local (intratumoral) or sustained delivery approaches, mainly because they have the potential for depot formation that underpins sustained, high-concentration local delivery for long periods of time with minimal systemic exposure and systemic toxicities. This is especially beneficial for unresectable solid tumors or for local treatment in the resection cavity to minimize recurrence. However, some serious considerations must be resolved before the translation of these studies to clinical practices.

*1) Absence of clinical trials for OV-hydrogel systems: *We affirm that there are no clinical trials reported, to the best of our knowledge, for a combination of OV-hydrogel as a reliable therapeutic strategy of cancer. The reason is that this field is relatively new and both modifying the biological (virus) and making changes to the delivery system (hydrogel) have regulatory challenges. To strengthen our claims regarding the hydrogel delivery system overcoming limitations of OVs, clinical trials are the critical next step required to validate these preclinical findings in humans. It is worth noting that hydrogel applications in other therapeutic contexts, including drug delivery systems, tissue engineering and regenerative medicine, vaccine adjuvants, and immunotherapy, depicts the established clinical safety and utility of hydrogels in clinical trials, contextualizing the potential of OV-hydrogel system to translate into clinical practices. *2) Safety and biocompatibility concerns:* Since most studies evaluated the pharmacokinetics of hydrogels and OV-carrying hydrogels in experimental environments and animal models, more careful analyses and precise characterizations are required for transferring these applications to human studies. The lack of clinical trials may lead to safety and biocompatibility considerations in the future. For instance, the byproducts of hydrogel degradation or the hydrogel materials themselves could elicit host immune responses, local inflammation, fibrosis, and toxicity, leading to reduced therapeutic efficacy. Controlling the degradation rate of hydrogels is also another important factor in eliciting immune responses. Too fast degradation can result in insufficient delivery of cargo, while too slow a degradation rate can augment inflammatory responses and fibrous encapsulation. These issues highlight the necessity of selecting biocompatible materials to minimize side effects and immune responses during long-term use. In this regard, using generally recognized as safe (GRAS) materials or materials such as gelatin, chitosan, and hyaluronic acid, whose biocompatibility is well established, could reduce side effects. Although the bioactivity and biodegradability of natural polymers give them an advantage over synthetic polymers, control over purity with stringent quality control during manufacturing and chemical modification can reduce their immunogenicity and allergenicity. *3) Manufacturing, scalability, and translational hurdles:* Other challenges in the OV-hydrogel system are manufacturing as well as translational hurdles. Maintaining batch-to-batch consistency under Good Manufacturing Practice (GMP) conditions and difficulties in scaling up hydrogels and OV-loading hydrogels, in addition to challenges of the sterilization process of biomaterials without affecting their structure and functions, are required to be addressed in future research to facilitate the translation of the OV-hydrogel system to clinical trials. During industrialization, large-scale production is a complex process that requires confirming the consistency of material properties and incorporating bioactive agents into the hydrogel system. *4) Cost and regulatory complexity:* The complex synthesis process and potentially high cost of raw biomaterials, besides the multifaceted processes of combining two distinct tools, hydrogel as a synthetic material and OV as a biological agent, require complex regulatory landscapes and safety testing. The future considerations should aim to eliminate cost through scalable, inexpensive alternatives such as alginate or scaled automated production platforms such as 3D bioprinting, that lessen labor and material costs. Regulatory simplification could take the form of creating standardized testing methods for hybrid systems, or providing similar use regulatory fast-track programs to orphan indications, assuring hydrogel-OV treats have a high economic viability and attain clinically navigable approval. *5) Hydrogel design optimization:* The determination of the hydrogel degradation rate and the factors that affect this parameter, such as hydrogel stiffness and cross-linking mode, will help to design more controllable micro/nanostructures to deliver OVs to tumor sites. Furthermore, modeling their release profile and targeting moieties could be helpful in achieving an ideal and personalized nano-delivery system for OVs using hydrogels. In this regard, machine learning (ML) and artificial intelligence (AI) models can analyze and predict the dynamics of both anti-tumor (e.g., CTLs) and anti-viral (e.g., neutralizing antibodies and NK cells) immune responses, the rate of tumor cell infection and lysis, the OV release kinetics and the hydrogel degradation rate. Analyzing both *in vitro* and *in vivo* results and predicting both OV and hydrogel behavior in the body using ML and AI models could accelerate the design of optimized OV-hydrogel formulations for personalized cancer therapy. Additionally, ML and AI could deal with the issues related to scalability and reproducibility by optimizing fabrication processes and material formulations. Taken together, interdisciplinary collaboration among genetic engineering, bioengineering, material science, computational modeling, and chemistry as well as long-term behavior of hydrogels could bridge the gaps and introduce the hydrogel system as an encouraging candidate for delivery of OVs and circumvent their limitations.

## Competing Interests

 The authors declare no conflict of interest.

## Data Availability Statement

 No data was used for the research described in the article.

## Ethical Approval

 Not applicable.

## References

[1] Hill C, Carlisle R (2019). Achieving systemic delivery of oncolytic viruses. Expert Opin Drug Deliv.

[2] Najafi M, Hashemi Goradel N, Farhood B, Salehi E, Solhjoo S, Toolee H (2019). Tumor microenvironment: interactions and therapy. J Cell Physiol.

[3] Chen DS, Mellman I (2017). Elements of cancer immunity and the cancer-immune set point. Nature.

[4] Ghanaat M, Hashemi Goradel N, Arashkia A, Ebrahimi N, Ghorghanlu S, Veisi Malekshahi Z (2021). Virus against virus: strategies for using adenovirus vectors in the treatment of HPV-induced cervical cancer. Acta Pharmacol Sin.

[5] Chen L, Ma Z, Xu C, Xie Y, Ouyang D, Song S (2023). Progress in oncolytic viruses modified with nanomaterials for intravenous application. Cancer Biol Med.

[6] Hemminki O, Dos Santos JM, Hemminki A (2020). Oncolytic viruses for cancer immunotherapy. J Hematol Oncol.

[7] Frampton JE (2022). Teserpaturev/G47Δ: first Approval. BioDrugs.

[8] Hashemi Goradel N, Baker AT, Arashkia A, Ebrahimi N, Ghorghanlu S, Negahdari B (2021). Oncolytic virotherapy: challenges and solutions. CurrProbl Cancer.

[9] Hashemi Goradel N, Negahdari B, Ghorghanlu S, Jahangiri S, Arashkia A (2020). Strategies for enhancing intratumoral spread of oncolytic adenoviruses. PharmacolTher.

[10] Choi JW, Kang E, Kwon OJ, Yun TJ, Park HK, Kim PH (2013). Local sustained delivery of oncolytic adenovirus with injectable alginate gel for cancer virotherapy. Gene Ther.

[11] Mashhadi Abolghasem Shirazi M, Saedi TA, Samadi Moghaddam Z, Nemati M, Shiri R, Negahdari B (2024). Nanotechnology and nano-sized tools: Newer approaches to circumvent oncolytic adenovirus limitations. PharmacolTher.

[12] Cao H, Duan L, Zhang Y, Cao J, Zhang K (2021). Current hydrogel advances in physicochemical and biological response-driven biomedical application diversity. Signal Transduct Target Ther.

[13] Mahmood A, Patel D, Hickson B, DesRochers J, Hu X (2022). Recent progress in biopolymer-based hydrogel materials for biomedical applications. Int J Mol Sci.

[14] Zhang X, Guo X, Wu Y, Gao J (2021). Locally injectable hydrogels for tumor immunotherapy. Gels.

[15] Qiao H, Chen X, Wang Q, Zhang J, Huang D, Chen E (2020). Tumor localization of oncolytic adenovirus assisted by pH-degradable microgels with JQ1-mediated boosting replication and PD-L1 suppression for enhanced cancer therapy. Biomater Sci.

[16] Xie Z, Shen J, Sun H, Li J, Wang X (2021). Polymer-based hydrogels with local drug release for cancer immunotherapy. Biomed Pharmacother.

[17] Kasiński A, Zielińska-Pisklak M, Oledzka E, Sobczak M (2020). Smart hydrogels - synthetic stimuli-responsive antitumor drug release systems. Int J Nanomedicine.

[18] Jafari M, Kadkhodazadeh M, Bahrololoumi Shapourabadi M, Hashemi Goradel N, Shokrgozar MA, Arashkia A (2022). Immunovirotherapy: the role of antibody-based therapeutics combination with oncolytic viruses. Front Immunol.

[19] Jung BK, Oh E, Hong J, Lee Y, Park KD, Yun CO (2017). A hydrogel matrix prolongs persistence and promotes specific localization of an oncolytic adenovirus in a tumor by restricting nonspecific shedding and an antiviral immune response. Biomaterials.

[20] Chen L, Zuo M, Zhou Q, Wang Y (2023). Oncolytic virotherapy in cancer treatment: challenges and optimization prospects. Front Immunol.

[21] Wang Z, Sun P, Li Z, Xiao S (2023). Clinical advances and future directions of oncolytic virotherapy for head and neck cancer. Cancers (Basel).

[22] Kaufman HL, Shalhout SZ, Iodice G (2022). Talimogene laherparepvec: moving from first-in-class to best-in-class. Front Mol Biosci.

[23] Aldrak N, Alsaab S, Algethami A, Bhere D, Wakimoto H, Shah K (2021). Oncolytic herpes simplex virus-based therapies for cancer. Cells.

[24] Hu M, Liao X, Tao Y, Chen Y (2023). Advances in oncolytic herpes simplex virus and adenovirus therapy for recurrent glioma. Front Immunol.

[25] Mantwill K, Klein FG, Wang D, Hindupur SV, Ehrenfeld M, Holm PS (2021). Concepts in oncolytic adenovirus therapy. Int J Mol Sci.

[26] Li K, Zhao Y, Hu X, Jiao J, Wang W, Yao H (2022). Advances in the clinical development of oncolytic viruses. Am J Transl Res.

[27] Blanchette P, Teodoro JG (2023). A renaissance for oncolytic adenoviruses?. Viruses.

[28] Hashemi Goradel N, Alizadeh A, Hosseinzadeh S, Taghipour M, Ghesmati Z, Arashkia A (2022). Oncolytic virotherapy as promising immunotherapy against cancer: mechanisms of resistance to oncolytic viruses. Future Oncol.

[29] Zhou YC, Zhang YN, Yang X, Wang SB, Hu PY (2020). Delivery systems for enhancing oncolytic adenoviruses efficacy. Int J Pharm.

[30] Wang LL, Burdick JA. Engineered hydrogels for local and sustained delivery of RNA-interference therapies. Adv Healthc Mater 2017;6(1):10.1002/adhm.201601041. doi: 10.1002/adhm.201601041. PMC5226889 27976524

[31] Schek RM, Hollister SJ, Krebsbach PH (2004). Delivery and protection of adenoviruses using biocompatible hydrogels for localized gene therapy. Mol Ther.

[32] Gustafson JA, Price RA, Greish K, Cappello J, Ghandehari H (2010). Silk-elastin-like hydrogel improves the safety of adenovirus-mediated gene-directed enzyme-prodrug therapy. Mol Pharm.

[33] Prasad A, Loh XJ. From bench to bedside—an example of an in-situ hydrogel in in vivo applications. In: Loh XJ, ed. In-Situ Gelling Polymers: For Biomedical Applications. Singapore: Springer; 2015. p. 215-26. doi: 10.1007/978-981-287-152-7_9.

[34] Truong CS, Yoo SY (2022). Oncolytic vaccinia virus in lung cancer vaccines. Vaccines (Basel).

[35] Mirbahari SN, Da Silva M, Zúñiga AIM, Kooshki Zamani N, St-Laurent G, Totonchi M (2024). Recent progress in combination therapy of oncolytic vaccinia virus. Front Immunol.

[36] Li M, Zhang M, Ye Q, Liu Y, Qian W (2023). Preclinical and clinical trials of oncolytic vaccinia virus in cancer immunotherapy: a comprehensive review. Cancer Biol Med.

[37] Deng L, Fan J, Ding Y, Zhang J, Zhou B, Zhang Y (2017). Oncolytic efficacy of thymidine kinase-deleted vaccinia virus strain Guang9. Oncotarget.

[38] Cousin S, Toulmonde M, Kind M, Guegan JP, Bessede A, Cantarel C (2022). Phase 2 trial of intravenous oncolytic virus JX-594 combined with low-dose cyclophosphamide in patients with advanced breast cancer. Exp Hematol Oncol.

[39] Engeland CE, Ungerechts G (2021). Measles virus as an oncolytic immunotherapy. Cancers (Basel).

[40] Scheicher NV. Oncolytic Virotherapy of Neuroendocrine Neoplasms with Oncolytic Measles Vaccine Virus [dissertation]. Tübingen: Universität Tübingen; 2024.

[41] Bucheit AD, Kumar S, Grote DM, Lin Y, von Messling V, Cattaneo RB (2003). An oncolytic measles virus engineered to enter cells through the CD20 antigen. Mol Ther.

[42] Eckhardt D, Bossow S, Klee JP, Boshof B, Ungerechts G, Czermak P, et al. Improved production strategies for oncolytic measles viruses as a therapeutic cancer treatment. In: Gautam S, Chiramel AI, Pach R, eds. Bioprocess and Analytics Development for Virus-based Advanced Therapeutics and Medicinal Products (ATMPs). Cham: Springer International Publishing; 2023. p. 375-405. doi: 10.1007/978-3-031-28489-2_16.

[43] Lal S, Raffel C (2017). Using cystine knot proteins as a novel approach to retarget oncolytic measles virus. Mol TherOncolytics.

[44] Ong HT, Trejo TR, Pham LD, Oberg AL, Russell SJ, Peng KW (2009). Intravascularly administered RGD-displaying measles viruses bind to and infect neovessel endothelial cells in vivo. Mol Ther.

[45] Leber MF, Neault S, Jirovec E, Barkley R, Said A, Bell JC (2020). Engineering and combining oncolytic measles virus for cancer therapy. Cytokine Growth Factor Rev.

[46] Galanis E, Dooley KE, Keith Anderson S, Kurokawa CB, Carrero XW, Uhm JH (2024). Carcinoembryonic antigen-expressing oncolytic measles virus derivative in recurrent glioblastoma: a phase 1 trial. Nat Commun.

[47] Noonan AM, Farren MR, Geyer SM, Huang Y, Tahiri S, Ahn D (2016). Randomized phase 2 trial of the oncolytic virus pelareorep (Reolysin) in upfront treatment of metastatic pancreatic adenocarcinoma. Mol Ther.

[48] Samson A, Scott KJ, Taggart D, West EJ, Wilson E, Nuovo GJ (2018). Intravenous delivery of oncolytic reovirus to brain tumor patients immunologically primes for subsequent checkpoint blockade. Sci Transl Med.

[49] Marchini A, Daeffler L, Pozdeev VI, Angelova A, Rommelaere J (2019). Immune conversion of tumor microenvironment by oncolytic viruses: the protoparvovirus H-1PV case study. Front Immunol.

[50] Geletneky K, Hajda J, Angelova AL, Leuchs B, Capper D, Bartsch AJ (2017). Oncolytic H-1 parvovirus shows safety and signs of immunogenic activity in a first phase I/IIa glioblastoma trial. Mol Ther.

[51] Hajda J, Leuchs B, Angelova AL, Frehtman V, Rommelaere J, Mertens M (2021). Phase 2 trial of oncolytic H-1 parvovirus therapy shows safety and signs of immune system activation in patients with metastatic pancreatic ductal adenocarcinoma. Clin Cancer Res.

[52] Beasley GM, Nair SK, Farrow NE, Landa K, Selim MA, Wiggs CA (2021). Phase I trial of intratumoral PVSRIPO in patients with unresectable, treatment-refractory melanoma. J Immunother Cancer.

[53] Brown MC, Holl EK, Boczkowski D, Dobrikova E, Mosaheb M, Chandramohan V (2017). Cancer immunotherapy with recombinant poliovirus induces IFN-dominant activation of dendritic cells and tumor antigen-specific CTLs. Sci Transl Med.

[54] Brown MC, Mosaheb MM, Mohme M, McKay ZP, Holl EK, Kastan JP (2021). Viral infection of cells within the tumor microenvironment mediates antitumor immunotherapy via selective TBK1-IRF3 signaling. Nat Commun.

[55] Sloan AE, Buerki RA, Murphy C, Kelly AT, Ambady P, Brown M (2021). LUMINOS-101: phase 2 study of PVSRIPO with pembrolizumab in recurrent glioblastoma. J Clin Oncol.

[56] Arnold D, Goekkurt E, Stein A, Martens UM, Chater J, Ungerechts G (2023). 1623P Pelareorep (pela) + atezolizumab (atezo) and chemotherapy in first-line (1L) advanced or metastatic pancreatic ductal adenocarcinoma (PDAC) patients: results from the GOBLET study. Ann Oncol.

[57] Sharma S, Tiwari S (2020). RETRACTED: a review on biomacromolecular hydrogel classification and its applications. Int J Biol Macromol.

[58] Ullah F, Othman MB, Javed F, Ahmad Z, Akil HM (2015). Classification, processing and application of hydrogels: a review. Mater Sci Eng C Mater Biol Appl.

[59] Ho TC, Chang CC, Chan HP, Chung TW, Shu CW, Chuang KP (2022). Hydrogels: properties and applications in biomedicine. Molecules.

[60] Ali F, Khan I, Chen J, Akhtar K, Bakhsh EM, Khan SB (2022). Emerging fabrication strategies of hydrogels and its applications. Gels.

[61] Zhang K, Xue K, Loh XJ (2021). Thermo-responsive hydrogels: from recent progress to biomedical applications. Gels.

[62] Darban Z, Shahabuddin S, Gaur R, Ahmad I, Sridewi N (2022). Hydrogel-based adsorbent material for the effective removal of heavy metals from wastewater: a comprehensive review. Gels.

[63] Zhang Y, Wu BM (2023). Current advances in stimuli-responsive hydrogels as smart drug delivery carriers. Gels.

[64] Khan B, Arbab A, Khan S, Fatima H, Bibi I, Chowdhry NP (2023). Recent progress in thermosensitive hydrogels and their applications in drug delivery area. MedCommBiomater Appl.

[65] Gu R, Zhou H, Zhang Z, Lv Y, Pan Y, Li Q (2023). Research progress related to thermosensitive hydrogel dressings in wound healing: a review. Nanoscale Adv.

[66] Chopra H, Singh I, Kumar S, Bhattacharya T, Rahman MH, Akter R (2022). A comprehensive review on hydrogels. Curr Drug Deliv.

[67] Hu Y, Shin Y, Park S, Jeong JP, Kim Y, Jung S (2022). Multifunctional oxidized succinoglycan/poly(N-isopropylacrylamide-co-acrylamide) hydrogels for drug delivery. Polymers (Basel).

[68] Wang X, Wang Q (2021). Enzyme-laden bioactive hydrogel for biocatalytic monitoring and regulation. Acc Chem Res.

[69] Sobczak M (2022). Enzyme-responsive hydrogels as potential drug delivery systems-state of knowledge and future prospects. Int J Mol Sci.

[70] Minehan RL, Del Borgo MP (2022). Controlled release of therapeutics from enzyme-responsive biomaterials. Front Biomater Sci.

[71] Wen Y, Wang X, Zhao J, Zhai X, Xia W, Li P (2025). Preparation and application of enzyme-based hydrogels. BiosensBioelectron X.

[72] Protsak IS, Morozov YM (2025). Fundamentals and advances in stimuli-responsive hydrogels and their applications: a review. Gels.

[73] Hasan N, Bhuyan MM, Jeong JH (2024). Single/multi-network conductive hydrogels-a review. Polymers (Basel).

[74] Pourjavadi A, Heydarpour R, Mazaheri Tehrani Z (2021). Multi-stimuli-responsive hydrogels and their medical applications. New J Chem.

[75] Roy A, Manna K, Pal S (2022). Recent advances in various stimuli-responsive hydrogels: from synthetic designs to emerging healthcare applications. Mater Chem Front.

[76] Cui L, Wang J, Liu M, Fan W, Sui K (2025). In situ growth of multiresponsive structural color patterns within hydrogels for multiple information encryption. ACS Appl Mater Interfaces.

[77] Ivanov AS, Pershina LV, Nikolaev KG, Skorb EV (2021). Recent progress of layer-by-layer assembly, free-standing film and hydrogel based on polyelectrolytes. MacromolBiosci.

[78] Yang P, Li Z, Fang B, Liu L (2023). Self-healing hydrogels based on biological macromolecules in wound healing: a review. Int J Biol Macromol.

[79] Ding X, Fan L, Wang L, Zhou M, Wang Y, Zhao Y (2023). Designing self-healing hydrogels for biomedical applications. Mater Horiz.

[80] Li B, Li C, Yan Z, Yang X, Xiao W, Zhang D (2025). A review of self-healing hydrogels for bone repair and regeneration: materials, mechanisms, and applications. Int J Biol Macromol.

[81] Rumon MM, Sayem M, Halder SK, Brishti RS, Das A, Hasan MA (2025). The promise of functionalized chitosan‐based self‐healing hydrogels. Adv Polym Technol.

[82] Qi YL, Zhou HY, Han GZ (2025). A novel photo-regulated self-healing hydrogel based on hollow SiO2@g-C3N4@TiO2 and PVA. RSC Adv.

[83] Rumon MM (2025). Advances in cellulose-based hydrogels: tunable swelling dynamics and their versatile real-time applications. RSC Adv.

[84] Kumar N, Singh S, Sharma P, Kumar B, Kumar A. Single-, dual-, and multi-stimuli-responsive nanogels for biomedical applications. Gels 2024;10(1). doi: 10.3390/gels10010061. PMC10815403 38247784

[85] Anooj ES, Charumathy M, Sharma V, Vibala BV, Gopukumar ST, Jainab SIB (2021). Nanogels: an overview of properties, biomedical applications, future research trends and developments. J Mol Struct.

[86] Wang Q, Zhang Y, Ma Y, Wang M, Pan G (2023). Nano-crosslinked dynamic hydrogels for biomedical applications. Mater Today Bio.

[87] Mastella P, Todaro B, Luin S (2024). Nanogels: recent advances in synthesis and biomedical applications. Nanomaterials (Basel).

[88] Zafaryab M, Vig K (2025). Biomedical application of nanogels: from cancer to wound healing. Molecules.

[89] Zhao Z, Sun F, Yu H, Li B, Wang X, Sun Y (2025). Nucleic acid-based nanogels for drug delivery: from construction strategies to intelligent applications. Colloids Surf B Biointerfaces.

[90] Pandey T, Sharma A, Sandhu A, Pandey V (2024). Controlled polymerization in microfluidics: advancement in nanogel synthesis. SPE Polym.

[91] Lauer UM, Beil J. Oncolytic viruses: challenges and considerations in an evolving clinical landscape. Future Oncol 2022. doi: 10.2217/fon-2022-0440. 35818970

[92] Zhao Y, Liu Z, Li L, Wu J, Zhang H, Zhang H (2021). Oncolytic adenovirus: prospects for cancer immunotherapy. Front Microbiol.

[93] Fu W, Guo M, Zhou X, Wang Z, Sun J, An Y (2024). Injectable hydrogel mucosal vaccine elicits protective immunity against respiratory viruses. ACS Nano.

[94] Roth GA, Gale EC, Alcántara-Hernández M, Luo W, Axpe E, Verma R (2020). Injectable hydrogels for sustained codelivery of subunit vaccines enhance humoral immunity. ACS Cent Sci.

[95] Jung SH, Choi JW, Yun CO, Yhee JY, Price R, Kim SH (2014). Sustained local delivery of oncolytic short hairpin RNA adenoviruses for treatment of head and neck cancer. J Gene Med.

[96] Amer LD, Bryant SJ (2016). The in vitro and in vivo response to MMP-sensitive poly(ethylene glycol) hydrogels. Ann Biomed Eng.

[97] Zhuang Z, Zhang Y, Sun S, Li Q, Chen K, An C (2020). Control of matrix stiffness using methacrylate-gelatin hydrogels for a macrophage-mediated inflammatory response. ACS Biomater Sci Eng.

[98] He H, Sun L, Ye J, Liu E, Chen S, Liang Q (2016). Enzyme-triggered, cell penetrating peptide-mediated delivery of anti-tumor agents. J Control Release.

[99] Rana MM, Demirkaya C, De la Hoz Siegler H (2024). Beyond needles: immunomodulatory hydrogel-guided vaccine delivery systems. Gels.

[100] Butenko S, Nagalla RR, Guerrero-Juarez CF, Palomba F, David LM, Nguyen RQ (2024). Hydrogel crosslinking modulates macrophages, fibroblasts, and their communication, during wound healing. Nat Commun.

[101] Le TM, Jung BK, Li Y, Duong HT, Nguyen TL, Hong JW (2019). Physically crosslinked injectable hydrogels for long-term delivery of oncolytic adenoviruses for cancer treatment. Biomater Sci.

[102] Deng S, Iscaro A, Zambito G, Mijiti Y, Minicucci M, Essand M (2021). Development of a new hyaluronic acid based redox-responsive nanohydrogel for the encapsulation of oncolytic viruses for cancer immunotherapy. Nanomaterials (Basel).

[103] Du YN, Wei Q, Zhao LJ, Fan CQ, Guo LR, Ye JF (2022). Hydrogel-based co-delivery of CIK cells and oncolytic adenovirus armed with IL12 and IL15 for cancer immunotherapy. Biomed Pharmacother.

[104] Oh E, Oh JE, Hong J, Chung Y, Lee Y, Park KD (2017). Optimized biodegradable polymeric reservoir-mediated local and sustained co-delivery of dendritic cells and oncolytic adenovirus co-expressing IL-12 and GM-CSF for cancer immunotherapy. J Control Release.

[105] Engelhart M, Lebiedz D, Sager S (2011). Optimal control for selected cancer chemotherapy ODE models: a view on the potential of optimal schedules and choice of objective function. Math Biosci.

[106] Barish S, Ochs MF, Sontag ED, Gevertz JL (2017). Evaluating optimal therapy robustness by virtual expansion of a sample population, with a case study in cancer immunotherapy. Proc Natl Acad Sci U S A.

[107] Cassidy T, Craig M (2019). Determinants of combination GM-CSF immunotherapy and oncolytic virotherapy success identified through in silico treatment personalization. PLoSComput Biol.

[108] Wahbi H, Ahmed E, Abalgaduir A, Alshammari F (2024). Mathematical model of cancer with ordinary differential equations. Contemp Math.

[109] Sharpe S, Dobrovolny HM (2021). Predicting the effectiveness of chemotherapy using stochastic ODE models of tumor growth. Commun Nonlinear Sci Numer Simul.

[110] Jarrett AM, Lima E, Hormuth DA 2nd, McKenna MT, Feng X, Ekrut DA (2018). Mathematical models of tumor cell proliferation: a review of the literature. Expert Rev Anticancer Ther.

[111] Jenner AL, Frascoli F, Yun CO, Kim PS (2020). Optimising hydrogel release profiles for viro-immunotherapy using oncolytic adenovirus expressing IL-12 and GM-CSF with immature dendritic cells. Appl Sci.

[112] Zhuang Q, Zhao B, Lin Z, Liang Y, Zhao Q, Wang Y (2023). Navoximod modulates local HSV-1 replication to reshape tumor immune microenvironment for enhanced immunotherapy via an injectable hydrogel. Commun Biol.

[113] Lv P, Chen H, Cheng H, Liu X, Liu C, Zeng Y (2023). A calcium alginate hydrogel microsphere‐based transcatheter arterial viroembolization strategy for hepatocellular carcinoma. Adv Ther.

